# Trimethylamine N-Oxide Exacerbates Renal Inflammation and Fibrosis in Rats With Diabetic Kidney Disease

**DOI:** 10.3389/fphys.2021.682482

**Published:** 2021-06-16

**Authors:** Qing Fang, Binjie Zheng, Na Liu, Jinfeng Liu, Wenhui Liu, Xinyi Huang, Xiangchang Zeng, Lulu Chen, Zhenyu Li, Dongsheng Ouyang

**Affiliations:** ^1^Department of Clinical Pharmacology, Xiangya Hospital, Central South University, Changsha, China; ^2^Hunan Key Laboratory of Pharmacogenetics, Institute of Clinical Pharmacology, Central South University, Changsha, China; ^3^Engineering Research Center of Applied Technology of Pharmacogenomics, Ministry of Education, Changsha, China; ^4^National Clinical Research Center for Geriatric Disorders, Changsha, China; ^5^Hunan Key Laboratory for Bioanalysis of Complex Matrix Samples, Changsha Duxact Biotech Co., Ltd., Changsha, China; ^6^Department of Geriatric Medicine, Xiangya Hospital, Central South University, Changsha, China

**Keywords:** trimethylamine N-oxide, diabetic kidney disease, inflammation, NLRP3, fibrosis

## Abstract

The gut microbiota plays a pivotal role in the onset and development of diabetes and its complications. Trimethylamine N-oxide (TMAO), a gut microbiota-dependent metabolite of certain nutrients, is associated with type 2 diabetes and its complications. Diabetic kidney disease (DKD) is one of the most serious microvascular complications. However, whether TMAO accelerates the development of DKD remains unclear. We tested the hypothesis that TMAO accelerates the development of DKD. A high-fat diet/low-dose streptozotocin-induced diabetes rat model was established, with or without TMAO in the rats’ drinking water. Compared to the normal rats, the DKD rats showed significantly higher plasma TMAO levels at the end of the study. TMAO treatment not only exacerbated the kidney dysfunction of the DKD rats, but also renal fibrosis. Furthermore, TMAO treatment activated the nucleotide-binding domain, leucine-rich-containing family, pyrin domain-containing-3 (NLRP3) inflammasome and resulted in the release of interleukin (IL)-1β and IL-18 to accelerate renal inflammation. These results suggested that TMAO aggravated renal inflammation and fibrosis in the DKD rats, which provides a new perspective to understand the pathogenesis of DKD and a potential novel target for preventing the progression of DKD.

## Introduction

Diabetic kidney disease (DKD), or diabetic nephropathy, is one of the most fatal complications of diabetes mellitus, and it is the most prevailing element of end-stage renal disease (Cansby et al., 2020). Metabolic changes caused by diabetes lead to proteinuria, progressive mesangial expansion, glomerular basement membrane thickening, tubulointerstitial fibrosis, and impaired renal function (Alicic et al., 2017). The underlying pathogenesis of DKD is complex and involves many different pathways. Studies have shown that several factors are major contributors in the pathophysiology of DKD, including oxidative stress, inflammation, overexpression of transforming growth factor-β_1_ (TGF-β_1_), and other metabolic alterations (Liu Y. et al., 2020; Pourheydar et al., 2020). Despite improved prognosis over the years, the pathogenesis of DKD has not been fully elucidated. Understanding the mechanism of DKD will enable prevention and early intervention, which will result in better outcomes.

The gut microbiota plays an important role in many diseases. Trimethylamine N-oxide (TMAO), which is a gut microbiota-dependent metabolite of L-carnitine, choline, and phosphatidylcholine (Wang et al., 2011), has been implicated in the pathogenesis of various human diseases, including metabolic disorders (Chen S. et al., 2019), cardiovascular disorders, and neurological disorders (Vogt et al., 2018). Many studies have revealed that TMAO levels are higher in people with diabetes than in healthy people (Winther et al., 2019). A number of clinical studies have also demonstrated a strong association between TMAO levels and diabetes mellitus (Dambrova et al., 2016; Shan et al., 2017; Dong et al., 2018). Moreover, TMAO levels are strongly associated with the degree of renal function (Missailidis et al., 2016; Stubbs et al., 2016), and increased TMAO levels can directly contribute to progressive renal fibrosis and dysfunction in animal models (Tang et al., 2015; Sun et al., 2017; Li et al., 2018). However, the roles and mechanisms of TMAO in DKD have not been elucidated.

The nucleotide-binding domain, leucine-rich-containing family, pyrin domain-containing-3 (NLRP3) inflammasome is an important factor in aggravating kidney inflammation and fibrosis by the processing and secretion of the pro-inflammatory cytokines interleukin (IL)-1β and IL-18 in DKD. Upon activation, the NLRP3 inflammasome promotes the secretion of IL-1β and IL-18, thereby contributing to the development of DKD (Li L. H. et al., 2019). Accumulating evidence from recent studies have suggested that renal NLRP3 is activated in DKD animal models, while the inhibition of its activity could reduce the inflammation of renal tissues and improve renal functions (Wang et al., 2017; Chen K. et al., 2019; Han et al., 2019). Furthermore, TMAO promotes the release of inflammatory factors by activating the NLRP3 inflammasome, thereby promoting vascular calcification, myocardial fibrosis, and vascular inflammation aggravating cardiovascular disease (Chen et al., 2017; Liu et al., 2019; Zhang et al., 2020). However, NLRP3 inflammasome in TMAO-mediated DKD remains unknown.

Here, we examined the effects of elevated TMAO levels on the development of DKD in diabetic rats. Investigating the effects and potential mechanisms of TMAO in DKD could provide a new perspective in the understanding of DKD.

## Materials and Methods

### Materials

TMAO was purchased from Aladdin Industrial Corporation (Shanghai, China). The purity of the TMAO, which was measured by high-performance liquid chromatography, was > 98%. Streptozotocin (STZ) was purchased from Beijing Solarbio Science & Technology Co., Ltd. (Beijing, China). Sodium citrate buffer (0.1 mol/L, pH 4.5, sterile) was also purchased from Beijing Solarbio Science & Technology Co., Ltd. (Beijing, China).

### Animals and Treatment

Adult male Sprague Dawley rats (*n* = 32) weighing 180–200 g were obtained from Hunan SJA Laboratory Animal Co., Ltd. (Hunan, China). All rats were maintained under specific pathogen-free conditions with a constant temperature of 23 ± 1°C and a dark-light cycle of 12:12 h. The study followed the National Guidelines for Laboratory Animal Welfare and was approved by the Experimental Animal Ethics Committee of Central South University (2019 sydw0208). All rats adapted to the laboratory environment for 7 days. They were then fed a normal diet or high-fat diet (HFD) and given drinking water with or without TMAO 0.2% (w/v) for 12 weeks, resulting in 4 experimental groups (*n* = 8 per group): CON, CON + TMAO, DKD, DKD + TMAO. For 4 weeks, the DKD groups were fed a HFD (Medicience, Ltd., Jiangsu, China) with the following composition: common breeding material, 63.5%; lard oil, 10%; sucrose, 20%; cholesterol, 2.5%; and sodium cholate, 0.5%. The DKD groups were then injected intraperitoneally with STZ at a low dose of 35 mg/kg diluted in citrate buffer. The control group was injected with citrate buffer. Diabetes was confirmed by measuring glucose levels after 72 h of the STZ injection. Rats with glucose levels ≥ 16.7 mmol/L were considered to be diabetic. The DKD groups continued to be fed the HFD for 8 weeks. All rats were put in metabolic cages for 24-h urine collection at the end of the 12 weeks. Blood samples were obtained by cardiac puncture at the time of euthanasia. Serum and plasma were then separated by centrifugation and stored at −80°C for subsequent experiments. In addition, the kidney was excised, weighed, and kept in liquid nitrogen or fixed in 4% paraformaldehyde.

### Biochemical Parameters Detection

The blood glucose levels of the rats were measured every 2 weeks after the STZ injection with glucose test strips (ACCU-CHEK, Shanghai, China). Total cholesterol (TC), triglyceride (TG), serum creatinine (Scr), and blood urea nitrogen (BUN) levels were detected by an automatic biochemical analyzer (Chemray 800, Shenzhen, China). Total 24-h urinary protein concentrations were detected with corresponding kits (Nanjing Jiancheng Bioengineering Institute, Nanjing, China).

### Circulating TMAO Measurements

TMAO levels were determined by measured by ultra-high-performance liquid chromatography-tandem mass spectrometry (UHPLC–MS) using d9-(trimethyl)-labeled internal standards as described previously (Jaworska et al., 2017).

### Histological Analysis

The renal tissues were fixed in 4% paraformaldehyde, embedded in paraffin, sectioned to a 5-μm thickness, and then stained with hematoxylin-eosin (H&E) and Masson stains for histological examination under a light microscope. Renal fibrosis was calculated based on the percentage of the collagen-positive area in the total tissue area (Tang et al., 2015). Tubular injury was graded from 0–4 based on the area of inflammatory cell infiltration, tubular epithelial cell atrophy, tubular vacuolization, and dilation region, as follows: 0%, 0; < 25%, 1; 25–50%, 2; 50–75%, 3; and > 75%, 4.

### Immunohistochemical Analysis

The expression of NLRP3 and caspase-1 in the renal tissue was detected by immunohistochemistry. All samples were fixed in 4% paraformaldehyde and embedded in paraffin. Then, the paraffin-embedded specimens were cut into 4-mm sections, deparaffinized, and rehydrated. Subsequently, the sections were placed in 3% H_2_O_2_ to eliminate endogenous peroxidase activity for 25 min. Next, the sections were blocked with normal goat serum, followed by incubation with 3% BSA for 30 min, followed by incubation with anti-NLRP3 (Affinity, DF7438, 1:100) and anti-caspase-1 (Abways, 1:100, CY5429) antibodies overnight at 4°C. After rinsing with phosphate-buffered saline, the sections were stained with a polymer horseradish peroxidase detection system (Servicebio, Beijing, China) and counterstained with hematoxylin. A total of 10 fields from each sample were randomly selected, and the positive-staining percentage was analyzed by Image-Pro Plus 6.0 software (Media Cybernetics).

### Enzyme Linked Immunosorbent Assay

The IL-1β in the renal tissue, IL-18 in the serum, and microalbumin in the urine (UAlb) were measured by enzyme-linked immunosorbent assay (ELISA). The kidney was cut into pieces, and the cut kidney tissue was prepared into its homogenate with 9 times the volume of normal saline. The homogenate was centrifuged at 3,500 r/min for 10 min to separate the supernatant, and the supernatant was preserved at 4°C for later use. The serum was centrifuged at 1,000 g for 15 min, and the supernatant was separated for later use. The IL-1β content in the renal tissue, the IL-18 content in the serum, and the microalbumin content in the urine were detected by ELISA according to kit instructions [MultiSciences (Lianke) Biotech Co., Ltd., Hangzhou, China; Cusabio Biotech Co., Ltd., Wuhan, China, CSB-E04610r; Cusabio Biotech Co., Ltd., Wuhan, China, CSB-E12991r].

### Western Blot Analysis

The kidney tissue was homogenized in mammalian protein extraction reagent lysis buffer (Merck Millipore, 92590) with protease inhibitor (NCM Biotech, P001). The supernatant was removed by centrifugation at 12,000 g for 15 min at 4°C. The total protein concentration was determined using the Micro BCA Protein Kit Assay (Pierce, Rockford, IL, United States). Protein from each sample (100 μg) was resolved by sodium dodecyl sulphate-polyacrylamide gel electrophoresis under reducing conditions, transferred to polyvinylidene fluoride membranes, and then blocked with 5% non-fat dry milk and 0.1% Tween-20 in Tris-buffered saline at room temperature for 2 h. Membranes were incubated overnight at 4°C with primary antibodies against GAPDH (Affinity, AF7021, 1:5,000), TGF-β1 (Affinity, AF1027, 1:1,000), α-SMA (Affinity, AF1032, 1:1,000), IL-1β (Affinity, AF5103, 1:1,000), and IL-18 (Affinity, DF6252, 1:1,000). After washing with Tris-buffered saline with Tween-20, membranes were incubated with a secondary goat anti-rabbit IgG horseradish peroxidase conjugate (1:5,000 dilution in secondary antibody dilution buffer) antibody (Affinity, S0001) or a secondary goat anti-rabbit IgG horseradish peroxidase conjugate (1:5,000 dilution in secondary antibody dilution buffer) antibody (Affinity, S0002) at room temperature for 1 h. Membranes were detected with a western blot detection system (WEST-ZOLR Plus, Intron Biotechnology, Shanghai, China) according to the manufacturer’s instructions and then exposed to X-ray film (Thermo Scientific, Shanghai, China). All experiments were repeated 3 times.

### Statistical Analyses

All statistical analyses were performed using the SPSS Statistics 22 software. GraphPad PRISM 8.0 (Vienna, Austria, 2018) was used to generate graphs. Data were expressed in terms of the mean ± SEM. Weights and blood glucose levels were analyzed using repeated measures analysis of variance (ANOVA). One-way ANOVA was used for comparisons between groups, and the least significant difference method was used to compare the variance among groups. The Spearman correlation was used to determine the associations between the circulating TMAO levels and other measured parameters. A *P* < 0.05 was considered statistically significant.

## Results

### Elevated Plasma TMAO Levels in DKD Rats

**FIGURE 1:**
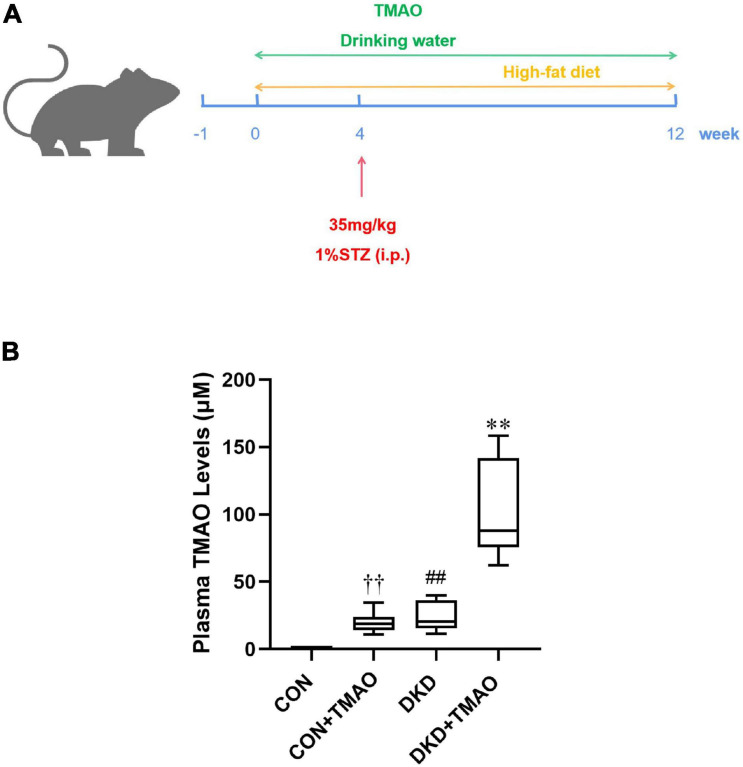
Plasma TMAO concentration in rats. **(A)** Schedule of 12-week experiment. The rats received streptozotocin (STZ) or citrate buffer by intraperitoneally (i.p.) at 4th week. Moreover, rats were treated with normal diet or high-fat-diet, with or without TMAO (0.2%) in drinking water. **(B)** Plasma TMAO levels in rats. Data are presented as mean ± SEM (*n* = 8 for each group). ^††^*P* < 0.01 vs. CON, ^##^*P* < 0.01 vs. CON, ^∗∗^*P* < 0.01 vs. DKD.

To analyze the effects of TMAO on DKD, we administered a HFD and intraperitoneal STZ injections to establish a diabetic rat model. The rats were given drinking water with or without TMAO for 12 weeks (Figure 1A). After 12 weeks of TMAO treatment, the plasma TMAO levels in the TMAO water-treated groups were elevated compared to those in the plain water-treated groups (CON, 0.91 ± 0.37 μM vs. CON + TMAO, 20.00 ± 7.03 μM; DKD, 24.01 ± 10.03 μM vs. DKD + TMAO 100.48 ± 34.36 μM). The TMAO levels in the DKD rats were significantly higher than those in the normal rats (Figure 1B).

### Effects of TMAO on Body Weight and Metabolic Parameters

**FIGURE 2:**
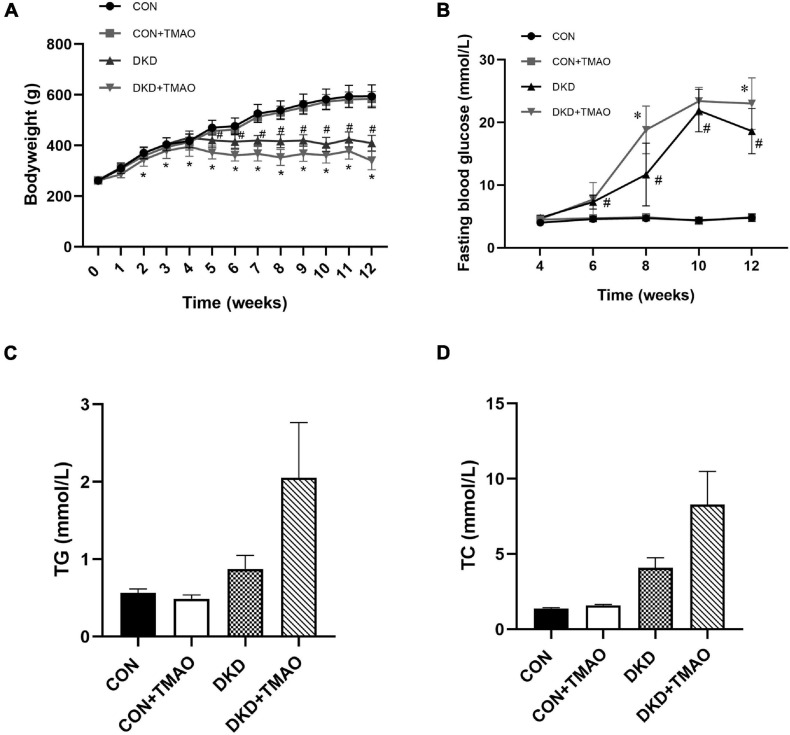
Body weight and metabolic parameters in rats. **(A)** Body weight, **(B)** Fasting blood glucose, **(C)** Cholesterol, **(D)** Triglyceride. Data are presented as mean ± SEM (*n* = 8 for each group). ^#^*P* < 0.05 vs. CON, ^∗^*P* < 0.05 vs. DKD.

The body weights were similar at baseline (Figure 2A). Compared to the rats in the CON group, the body weights of the rats in the DKD group were decreased from the fifth week and the fasting blood glucose levels of the rats in the DKD group were higher from the sixth week. There were no body weight or fasting blood glucose differences between the CON and CON + TMAO groups. The body weights of the rats in the DKD + TMAO group were decreased compared to those of the rats in the DKD group from the third week (Figure 2A). The fasting blood glucose levels of the rats in the DKD + TMAO group were higher than those of the rats in the DKD group at the eighth and twelfth weeks (Figure 2B). Compared to the CON group, the serum TC and TG levels were not significantly higher in the DKD group. Further, there were no differences in the serum TC and TG levels between the DKD and DKD + TMAO groups (Figures 2C,D).

### Effects of TMAO on Renal Function

**FIGURE 3:**
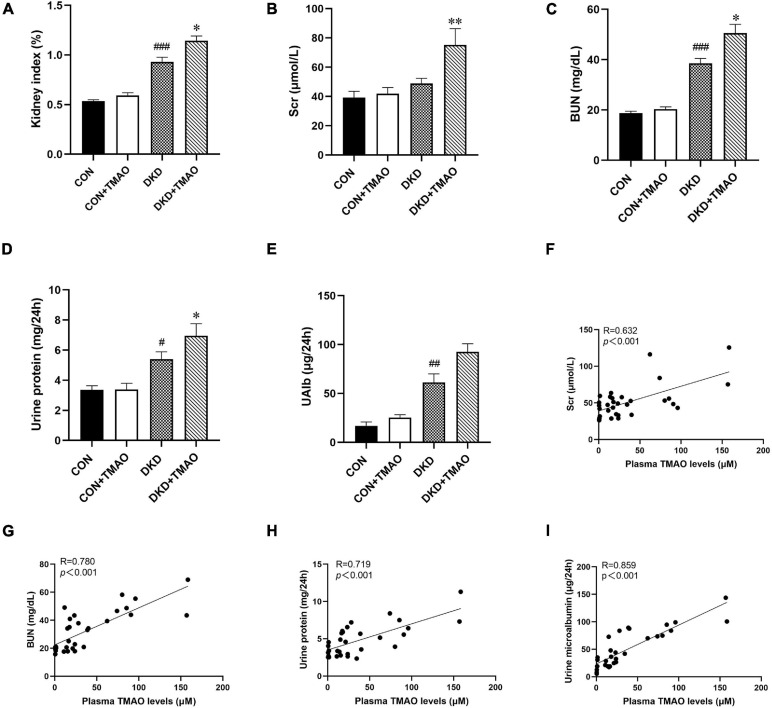
Parameters of renal function in rats. **(A)** Kidney index (kidney weight/body weight), **(B)** Scr, **(C)** BUN, **(D)** Urine protein, **(E)** Urine microalbumin. Plasma TMAO levels were significantly correlated with renal function parameters Scr **(F)**, BUN **(G)**, Urine protein **(H)**, and Urine microalbumin **(I)**. Data are presented as mean ± SEM (*n* = 8 for each group). ^#^*P* < 0.05, ^##^*P* < 0.01, ^###^*P* < 0.001 vs. CON, ^∗^*P* < 0.05, ^∗∗^*P* < 0.01 vs. DKD.

Compared to the CON group, the kidney index, BUN, urine protein, and UAlb levels were significantly higher in the DKD group. There were no differences in these parameters between the CON and CON + TMAO groups. The kidney index, Scr, BUN, and urine protein levels were significantly higher in the DKD + TMAO group compared to those in the DKD group (Figures 3A–E). Importantly, the plasma TMAO levels were positively correlated with the renal function parameters of Scr, BUN, urine protein, and UAlb levels (Figures 3F–I).

### Effects of TMAO on Renal Histopathological Changes

**FIGURE 4:**
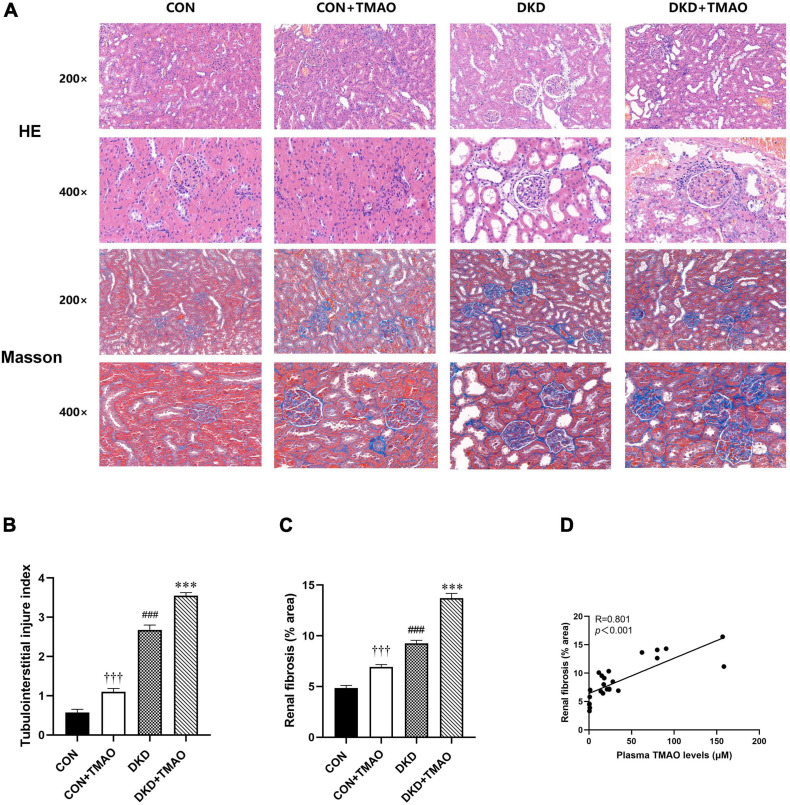
Pathology results in kidney of rats. **(A)** The results of HE staining and Masson staining in all rat groups (enlargement factor: 200×, 400×). **(B)** Tubulointerstitial injury index. **(C)** Fibrosis area of Masson staining. **(D)** The relationship between plasma TMAO levels and renal fibrosis area. Data are presented as mean ± SEM (*n* > 6 for each group). ^†††^*P* < 0.001 vs. CON, ^###^*P* < 0.001 vs. CON, ^∗∗∗^*P* < 0.001 vs. DKD.

The H&E staining results showed that inflammatory cell infiltration, tubular dilatation, cavitation of distal convoluted tubules, and tubular atrophy were present in the DKD group compared to the CON group. In contrast to the CON group, a small amount of inflammatory cell infiltration in the tubular interstitium and slight tubular dilation were present in the rats in the CON + TMAO group. The DKD + TMAO group experienced more serious renal pathological alterations compared to the DKD group (Figures 4A,B). The Masson staining results showed that a larger fibrosis area was present in the rats in the DKD group compared to the rats in the CON group. In contrast to the rats in the CON group, the fibrosis area in the rats in the CON + TMAO group was significantly increased. Meanwhile, the fibrosis area in the DKD rats was significantly increased after the TMAO intervention (Figures 4A,C). Furthermore, the plasma TMAO levels were positively correlated with renal fibrosis area (Figure 4D).

### Effects of TMAO on the Expression of TGF-β_1_ and α-SMA

**FIGURE 5:**
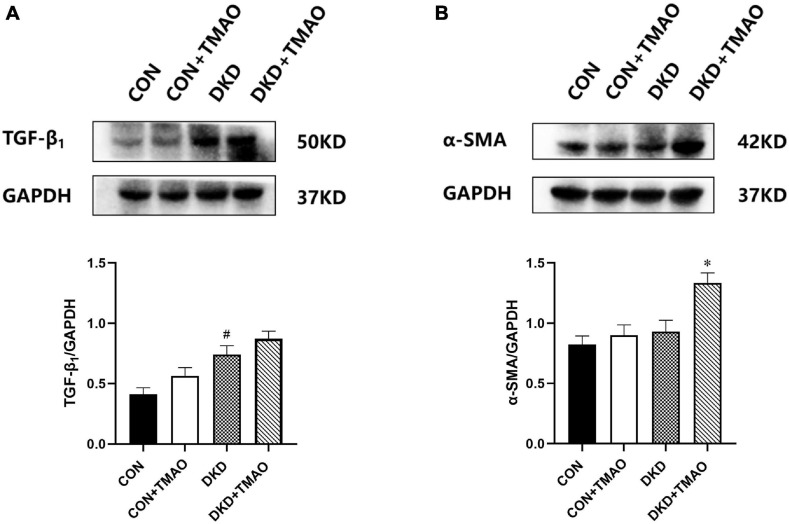
Expression of renal fibrosis proteins in the kidney of rats. **(A)** TGF-β_1_ protein levels. **(B)** α-SMA protein levels. Data are presented as mean ± SEM (*n* > 3 for each group). ^#^*P* < 0.05 vs. CON, ^∗^*P* < 0.05 vs. DKD.

To further examine whether elevated plasma TMAO levels contributed to renal fibrosis, we measured the expression of TGF-β_1_ and α-SMA in the kidney tissue by western blot analysis (Figure 5). The results showed that the expression of TGF-β_1_ was significantly increased in the kidney tissue of the rats in the DKD group compared to that of the rats in the CON group (Figure 5A). There was no significant difference in the expression of TGF-β_1_ or α-SMA between the CON and CON + TMAO groups (Figures 5A,B). The expression of α-SMA was significantly increased in the kidney tissue of the rats in the DKD + TMAO group compared to that of the rats in the DKD group (Figure 5B). However, the expression of TGF-β_1_ was not significantly increased in the kidney tissue of the rats in the DKD + TMAO group (Figure 5A).

### Effects of TMAO on Renal Inflammation

**FIGURE 6:**
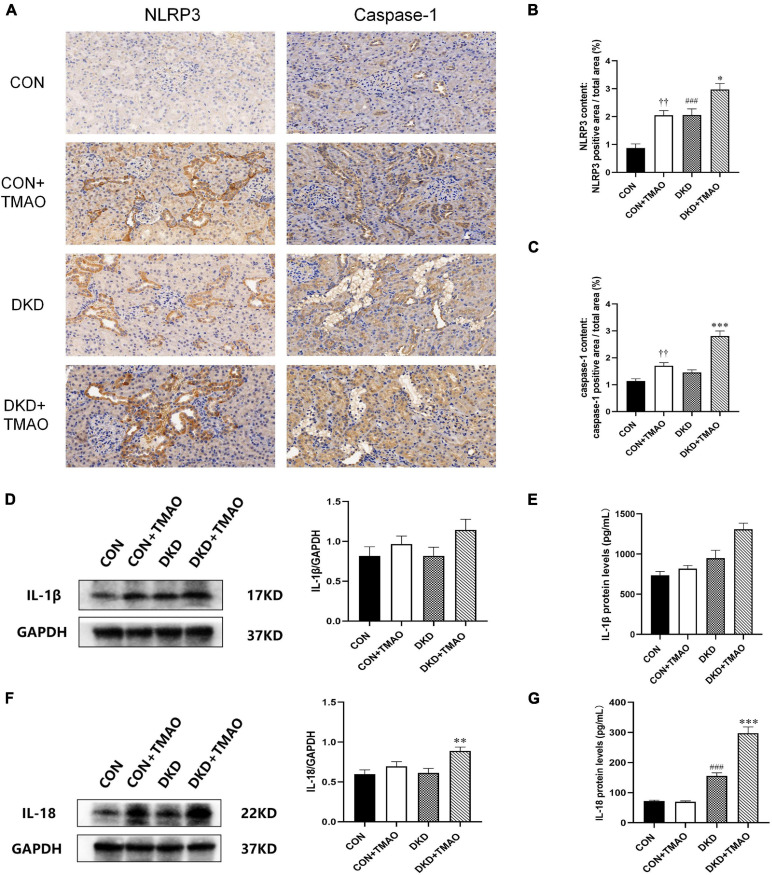
Immunohistochemistry, Western blot and ELISA results of NLRP3 inflammasome in rats. **(A)** Immunohistochemistry results of NLRP3 and caspase-1 in rats. Relative percentages of **(B)** NLRP3 and **(C)** caspase-1 positive area to total area. **(D)** Western blot and **(E)** ELISA results of IL-1β in kidney of rats. **(F)** Western blot and **(G)** ELISA results of IL-18 in kidney and serum of rats. Data are presented as mean ± SEM (*n* = 3–8 for each group). ^††^*P* < 0.01 vs. CON, ^###^*P* < 0.001 vs. CON, *P < 0.05, **P < 0.01, ***P < 0.001 vs. DKD.

The NLRP3 inflammasome signaling pathway, including NLRP3, caspase-1, IL-1β, and IL-18, was evaluated using immunohistochemistry, western blot analysis, and ELISA in order to explore the presumed mechanism of the pro-inflammatory activity of TMAO (Figure 6). The results showed that the levels of NLRP3 and IL-18 were significantly increased in the DKD group compared to the CON group (Figures 6B,G). Compared to the DKD group, the levels of NLRP3, caspase-1, IL-1β, and IL-18 were all significantly increased in the DKD + TMAO group (Figures 6B–G). Further, the levels of NLRP3 and caspase-1 in the CON + TMAO group were significantly increased compared to those in the CON group (Figures 6B,C).

## Discussion

**FIGURE 7:**
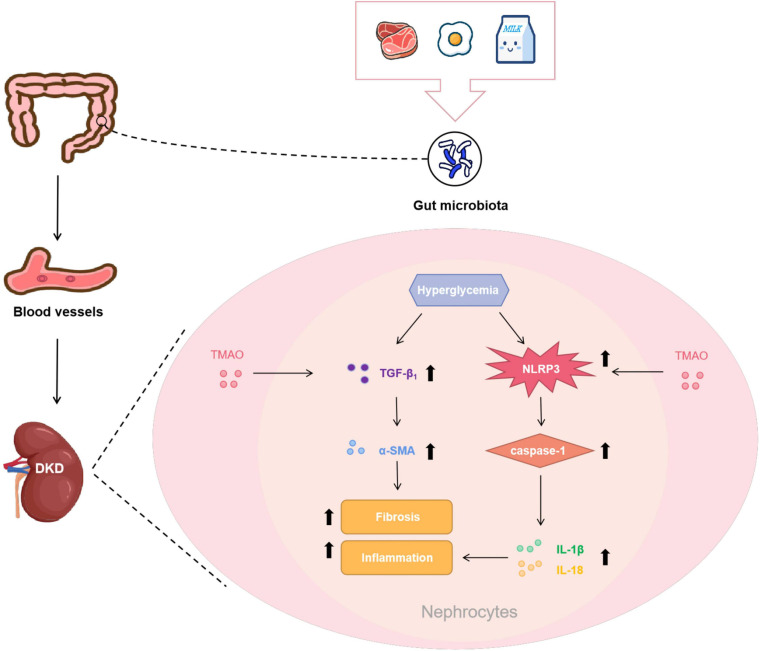
Potential mechanisms of pro-DKD effect of TMAO. Foods like red meat, eggs and milk are digested and absorbed by the gastrointestinal tract, subsequently metabolized under the action of gut microbiota and then oxidized by the liver to form TMAO to enter the bloodstream. TMAO entered the kidney tissue with the blood, and facilitated renal fibrosis by increasing pro-fibrotic factor TGF-β_1_ and its downstream molecule α-SMA. TMAO may also activated the NLRP3 inflammasome to cause the release of IL-1β and IL-18 to promote renal inflammation. These effects together aggravated the progression of DKD.

The major findings of this study were: (1) the rats with DKD had increased circulating TMAO levels; (2) the circulating TMAO levels of the CON + TMAO rats administered TMAO for 12 weeks were almost the same as those of the DKD rats; (3) TMAO administration in the DKD group decreased the body weights and increased the fasting blood glucose levels of the rats, while it had no effect on TG and TC levels; and (4) TMAO facilitated tubulointerstitial injury and renal fibrosis, and it activated the NLRP3 inflammasome to exacerbate renal inflammation. Collectively, these findings suggest that elevated TMAO levels exacerbate renal fibrosis and renal inflammation to accelerate the development of DKD (Figure 7).

More and more clinical evidence supports the close relationship between elevated plasma TMAO levels and increased risk of cardiovascular disease risk (Kanitsoraphan et al., 2018; Yang S. et al., 2019). Elevated TMAO levels also appear in many other diseases, including diabetes, chronic kidney disease, non-alcoholic fatty liver disease, and neurodegenerative diseases (Pelletier et al., 2019; Tan et al., 2019; Zhao L. et al., 2019; Zhuang et al., 2019). We previously found that people with DKD had higher TMAO levels than healthy people. In this study, we found that the plasma TMAO levels of the DKD rats were significantly higher than those of the normal rats. Most previous animal experiments have increased the levels of TMAO in the animals by adding 1% choline or 0.12% TMAO to their diets (Tang et al., 2015; Zhu et al., 2016). Adding TMAO at a concentration of 333 mg/L to the animals’ drinking water or administering TMAO by gavage at a dose of 120 mg/kg/day can also increase the levels of TMAO in the animals (Huc et al., 2018; Zhao Z. H. et al., 2019). In this study, we dissolved TMAO in the rats’ drinking water for 12 weeks. The plasma TMAO levels in the CON + TMAO group (20.00 ± 7.03 μM) increased to about 22 times those in the normal control group (0.91 ± 0.37 μM). The plasma TMAO levels of the rats with diabetic nephropathy were similar (24.01 ± 10.03 μM). Thus, administering male Sprague Dawley rats a 0.2% TMAO solution for 12 weeks can effectively increase their plasma TMAO levels to a level equivalent to the pathological state of DKD. This result can provide a reference for TMAO dose selection in the study of TMAO and diabetes or its complications.

In recent years, a growing body of evidence has demonstrated that the gut microbiota plays a pivotal role in the onset and development of diabetes and its complications. TMAO, a gut microbiota-dependent metabolite from foods such as red meat, eggs, and fish (Cho et al., 2017), is associated with type 2 diabetes and its complications (Dambrova et al., 2016; Shan et al., 2017; Liu W. et al., 2020). Furthermore, diet-induced changes in TMAO and its precursors are significantly associated with improvements in glycemia and insulin sensitivity (Heianza et al., 2019). Higher TMAO concentrations can impair glucose tolerance in HFD-fed mice (Gao et al., 2014, 2015). In the present study, we found that TMAO significantly increased the fasting blood glucose levels of the diabetic rats, but it did not affect the normal rats, while other studies have found that TMAO had no significant effect on fasting glucose levels in HFD-fed mice (Gao et al., 2014, 2015). The discrepancy may result from many factors, such as the administration of TMAO with STZ and the long-term treatment with TMAO in our study. Lipid metabolism disturbance is a pathogenic factor associated with DKD, with increased TC and TG levels in people and animals with DKD (Alaofi, 2020; Liu L. et al., 2020). The lipid metabolism disorder caused by TMAO has been proven in a number of animal experiments. A high-cholinergic diet can significantly increase the serum total TC and TG levels in mice fed a HFD (Yang C. et al., 2020). Furthermore, serum TG and TC levels have been shown to increase after intraperitoneal injection of TMAO in rats (Liu et al., 2021). In this study, we detected the serum TC and TG concentrations in the rats. The results showed that the serum TC and TG concentrations of the DKD + TMAO group were higher than those of the DKD group, but the difference was not statistically significant, which may be due to the large difference within the group. In our follow-up study, we may further expand the sample size to verify the effect of TMAO on lipid metabolism.

Accumulating evidence has also shown that increased TMAO levels are associated with a risk for all-cause mortality, and increased TMAO levels have been identified as an independent predictor of mortality in patients with chronic kidney disease (CKD) (Tang et al., 2015; Burdmann et al., 2016). We previously demonstrated in Chinese patients with CKD that combinations of TMAO and its precursors were related to glomerular filtration rate, which is an indicator of kidney function (Guo et al., 2020). Some animal studies suggest that elevated TMAO levels may directly impair renal function by contributing to oxidative stress, endothelial dysfunction, renal fibrosis, and other mechanisms (Sun et al., 2017; Li et al., 2018). In this study, we found that in the process of DKD, the renal function parameters of renal index, Scr, BUN, urine protein, and UAlb concentrations were increased after TMAO administration, although there was no significant difference in UAlb between the two groups. Pearson correlation analysis results showed that the plasma TMAO levels were positively correlated with Scr, BUN, 24-h urinary total protein, and UAlb levels, and the H&E staining results showed that TMAO further aggravated the degree of renal tubular damage. Taken together, we can infer that TMAO can promote reduced renal function, which may be a risk factor for DKD.

Renal fibrosis plays an important role in the development of DKD. It is an irreversible pathological change and the final and only common pathway for DKD to progress to end-stage renal disease (Zeng et al., 2019). Epithelial-to-mesenchymal transition is the main pathological process of renal interstitial fibrosis, and it is the initial step of renal fibrosis. It refers to the transformation of renal tubular epithelial cells into mesenchymal cells, and it can increase the expression of α-SMA, the marker protein of mesenchymal cells (Zhang et al., 2017). Renal interstitial fibrosis is also regulated by a variety of pro-fibrotic factors, among which TGF-β_1_, which plays an important role in renal interstitial fibrosis, is a very important regulatory factor. The expression of TGF-β_1_ mRNA and the TGF-β_1_ protein in the kidney tissue of people with diabetes is increased (Zhang et al., 2017). TGF-β_1_ is directly involved in the epithelial-to-mesenchymal transition process of DKD, leading to renal interstitial fibrosis (Zheng et al., 2016; Yang Y. et al., 2020), and it can activate the downstream Smad signaling pathway, thereby mediating fibrogenesis (Hathaway et al., 2015). The role of TMAO in promoting fibrosis has also been demonstrated in many studies. TMAO can activate the TGF-β receptor type I/Smad2 pathway, increase the expression of α-SMA and type I collagen, and promote the induction of cardiac fibrosis (Yang W. et al., 2019). In addition, TMAO can promote cardiac fibrosis by activating the Smad3 pathway in Sprague Dawley rats (Huang et al., 2018). TMAO can also aggravate Adriamycin-induced cardiac fibrosis in mice by activating the TGF-β/Smad3 pathway (Liu et al., 2019). Moreover, the TMAO inhibitor 3,3-dimethyl-1-butanol (DMB) can inhibit the TGF-β_1_/Smad3 pathway by reducing TMAO levels, thereby alleviating cardiac fibrosis in mice (Wang et al., 2020). Iodomethylcholine can inhibit the progression of adenine-induced CKD in mice by inhibiting TMAO levels and reducing collagen deposition (Zhang et al., 2021), and it can also inhibit the expression of pro-fibrotic genes, such as those encoding for TGF-β, type I collagen, tissue inhibitor of metalloproteinase 1, and α-SMA, to alleviate renal tubular interstitial fibrosis and dysfunction in CKD mice (Gupta et al., 2020). In this study, the Masson staining results directly showed renal interstitial fibrosis in the rats with DKD, and the degree of renal interstitial fibrosis in the DKD + TMAO group was further aggravated. More interestingly, we observed slight renal interstitial fibrosis in the normal rats administered TMAO. Through the detection of renal interstitial fibrosis-related proteins, we found that the expression of α-SMA increased significantly in the DKD rats after TMAO treatment, and the expression of TGF-β_1_ also increased, but there was no statistically significant difference. These results suggested that TMAO could promote renal interstitial fibrosis in the rats with DKD and that TMAO could slightly adversely affect the kidneys of the normal rats, but the specific mechanism of TMAO in promoting fibrosis needs to be further explored.

Emerging evidence suggests that inflammation plays a key role in the DKD progression (Moreno et al., 2018). Numerous preclinical studies have shown that several anti-inflammatory molecules can effectively improve DKD (Al Hroob et al., 2018; Olatunji et al., 2018; Feng et al., 2019). Interestingly, accumulating studies have shown that TMAO accelerates the progression of many inflammatory diseases, including cardiovascular disease, CKD, and central nervous system disease, by activating inflammatory pathways, such as the MAPK, NF-κB, and NLRP3 signaling pathways, and then increasing pro-inflammatory molecules, including tumor necrosis factor alpha, IL-6, IL-1β, and IL-18 (Seldin et al., 2016; Sun et al., 2017; Geng et al., 2018; Zhang et al., 2019, 2020). The NLRP3 inflammasome, which comprises different domains, such as NLRP3, ASC, caspase-1, IL-1β, and IL-18, has been shown to have a crucial role in DKD (Ram et al., 2020), and to be related to renal inflammation and fibrosis (Alzahrani et al., 2020; An et al., 2020). One study has shown that NLRP3 knockout in diabetic mice protects against diabetic nephropathy, improves the urine albumin/creatinine ratio, and attenuates glomerular hypertrophy, mesangial expansion, interstitial fibrosis, inflammation, and TGF-β_1_ expression (Wu et al., 2018). Recently, several studies have shown that TMAO exacerbates cardiac fibrosis, vascular calcification, and endothelial dysfunction by activating the NLRP3 inflammasome (Boini et al., 2017; Li X. et al., 2019; Zhang et al., 2020). In the present study, we demonstrated that TMAO could increase the expression of NLRP3, caspase-1, IL-1β, and IL-18 in the kidney of DKD rats, while TMAO could also significantly increase the protein levels of NLRP3 and caspase-1 in the kidney of normal rats. Thus, we speculate that TMAO may activate the NLRP3 inflammasome to aggravate renal inflammation to facilitate the development of DKD.

This study has some limitations. First, this study used the method of adding TMAO to increase the TMAO levels in the animals in order to explore the effects of TMAO on the disease, which was also the method used in many similar studies (Wang et al., 2011; Tang et al., 2015; Zhu et al., 2016). In the future, we intend to use TMAO inhibitors, such as DMB and iodomethylcholine, to further explore whether TMAO can be a therapeutic target for DKD. Finding new compounds that can inhibit TMAO levels is also an important research goal. Second, many studies have suggested that inflammation is an important mechanism for TMAO to promote the occurrence and development of diseases (Yang G. et al., 2019). In addition to the NLRP3 inflammasome in this study, it is also necessary to further explore whether TMAO promotes the progression of DKD through other inflammatory pathways, such as the NF-κB, MAPK, etc. pathways. Finally, several recent studies have reported that TMAO can directly increase the production of reactive oxygen species (Govindarajulu et al., 2020; Wu et al., 2020; Chang et al., 2021). The excessive production of reactive oxygen species caused by oxidative stress plays an important role in the pathogenesis of DKD (Jha et al., 2016). At the same time, reactive oxygen species are also a risk factor to activate the NLRP3 inflammasome (Han et al., 2018). Whether TMAO can directly activate oxidative stress to promote the progression of DKD remains to be further explored.

In conclusion, the results of our study can help improve our understanding of DKD by providing a novel mechanistic link between TMAO and DKD. We demonstrated that TMAO promotes renal inflammation and fibrosis in DKD rats. In addition, we found that NLRP3 inflammasome-mediated renal inflammation may be an important mechanism for TMAO to facilitate DKD. These findings may provide new insights into the mechanisms underlying DKD. Targeting TMAO may be a novel strategy for the prevention and treatment of DKD.

## Data Availability Statement

The raw data supporting the conclusions of this article will be made available by the authors, without undue reservation.

## Ethics Statement

The animal study was reviewed and approved by the Experimental Animal Ethics Committee of the central south University.

## Author Contributions

QF, BZ, and NL conceived and designed the experiments. QF, BZ, NL, WL, and JL performed the experiments. QF, XH, and XZ analyzed the data. QF, LC, ZL, and DO wrote the manuscript. All authors contributed to the article and approved the submitted version.

## Conflict of Interest

QF, BZ, NL, JL, WL, XH, XZ, LC, and DO were employed by the company Changsha Duxact Biotech Co., Ltd. The remaining author declares that the research was conducted in the absence of any commercial or financial relationships that could be construed as a potential conflict of interest.
